# Symbiotic nutrient exchange enhances the long-term survival of cassiosomes, the autonomous stinging-cell structures of *Cassiopea*

**DOI:** 10.1128/msphere.00322-23

**Published:** 2023-12-13

**Authors:** Gaëlle Toullec, Niclas Heidelberg Lyndby, Guilhem Banc-Prandi, Claudia Pogoreutz, Cristina Martin Olmos, Anders Meibom, Nils Rädecker

**Affiliations:** 1 Laboratory for Biological Geochemistry, School of Architecture, Civil and Environmental Engineering, École Polytechnique Fédérale de Lausanne (EPFL), Lausanne, Switzerland; 2 PSL Université Paris: EPHE-UPVD-CNRS, UAR 3278 CRIOBE, Université de Perpignan, Perpignan, France; 3 Center for Advanced Surface Analysis, Institute of Earth Science, University of Lausanne, Lausanne, Switzerland; Clemson University, Clemson, South Carolina, USA

**Keywords:** upside-down jellyfish, photosymbiosis, metabolism, Rhizostomae, stable isotope labeling, cryo-SEM, NanoSIMS

## Abstract

Medusae of the widely distributed upside-down jellyfish *Cassiopea* release autonomous, mobile stinging structures. These so-called cassiosomes play a role in predator defense and prey capture, and are major contributors to “contactless” stinging incidents in (sub-)tropical shallow waters. While the presence of endosymbiotic dinoflagellates in cassiosomes has previously been observed, their potential contribution to the metabolism and long-term survival of cassiosomes is unknown. Combining stable isotope labeling and correlative scanning electron microscopy and nanoscale secondary ion mass spectrometry imaging with a long-term *in vitro* experiment, our study reveals a mutualistic symbiosis based on nutritional exchanges in dinoflagellate-bearing cassiosomes. We show that organic carbon input from the dinoflagellates fuels the metabolism of the host tissue and enables anabolic nitrogen assimilation. This symbiotic nutrient exchange enhances the life span of cassiosomes for at least one month *in vitro*. Overall, our study demonstrates that cassiosomes, in analogy with *Cassiopea* medusae, are photosymbiotic holobionts. Cassiosomes, which are easily accessible under aquarium conditions, promise to be a powerful new miniaturized model system for in-depth ultrastructural and molecular investigation of cnidarian photosymbioses.

**IMPORTANCE:**

The upside-down jellyfish *Cassiopea* releases autonomous tissue structures, which are a major cause of contactless stinging incidents in (sub-) tropical coastal waters. These so-called cassiosomes frequently harbor algal symbionts, yet their role in cassiosome functioning and survival is unknown. Our results show that cassiosomes are metabolically active and supported by algal symbionts. Algal photosynthesis enhances the cassiosomes long-term survival in the light. This functional understanding of cassiosomes thereby contributes to explaining the prevalence of contactless stinging incidents and the ecological success of some *Cassiopea* species. Finally, we show that cassiosomes are miniaturized symbiotic holobionts that can be used to study host-microbe interactions in a simplified system.

## INTRODUCTION

Jellyfish (scyphomedusae) blooms can have significant impacts on marine ecosystems (1–3) and the human communities that depend on them (4, 5). Beyond their role in the marine food web (3), in biogeochemical cycling (1, 2), and in fisheries (5), jellyfish blooms also lead to increases in sting-related injuries to swimmers (4). Because of this stinging threat, jellyfish blooms thus have a strong negative effect on coastal tourism (3–6). Jellyfish blooms have been linked to the rise of sea surface temperatures and other human disturbances, such as eutrophication and overfishing (4, 7). The frequency and extent of these blooms have thus been predicted to locally increase or strongly oscillate in the future (7–10).

Some species of the jellyfish genus *Cassiopea* (Scyphozoa, Rhizostomae) have recently been reported as newly introduced and locally invasive in numerous localities (11–13). Due to their relatively high heat tolerance and trophic plasticity, their population density and geographic expansion are only expected to increase further (14–17). Like all cnidarians, *Cassiopea* medusae harbor specialized stinging cells called nematocytes that play an important role in predator defense and prey capture. While *Cassiopea* stings are often considered mild, Muffet et al. (18) recently highlighted their potential severity and a lack of public awareness regarding their threat. Jellyfish stings by direct contact are well known, but “contactless” stinging without direct physical contact with the animal has also been reported (18). Among contactless stinging mechanisms, the release of cassiosomes (i.e., autonomous, stinging, and often motile tissue structures) has been recently described in several rhizostome medusae (19), including some *Cassiopea* species (19–21). Interestingly, the cassiosomes from *Cassiopea xamachana, Cassiopea ornata,* and two Mastigiidae medusae host phototrophic dinoflagellates of the Symbiodiniaceae family, a group known to form endosymbiotic relationships with a diversity of cnidarians, such as corals and sea anemones (19, 20, 22–24). *Cassiopea* symbiotic medusae and the polyps (after uptake of Symbiodiniaceae) benefit strongly from organic carbon input from their dinoflagellate symbionts and are now well-established model systems for the cnidarian-Symbiodiniaceae symbiosis (25–29). However, the contribution of dinoflagellates to the metabolism and survival of cassiosomes remains unknown. Disentangling the metabolic activity and survival capacity of cassiosomes is therefore a key step to understanding and predicting the stinging threat represented by these cassiosomes in the marine environment.

**FIG 1:**
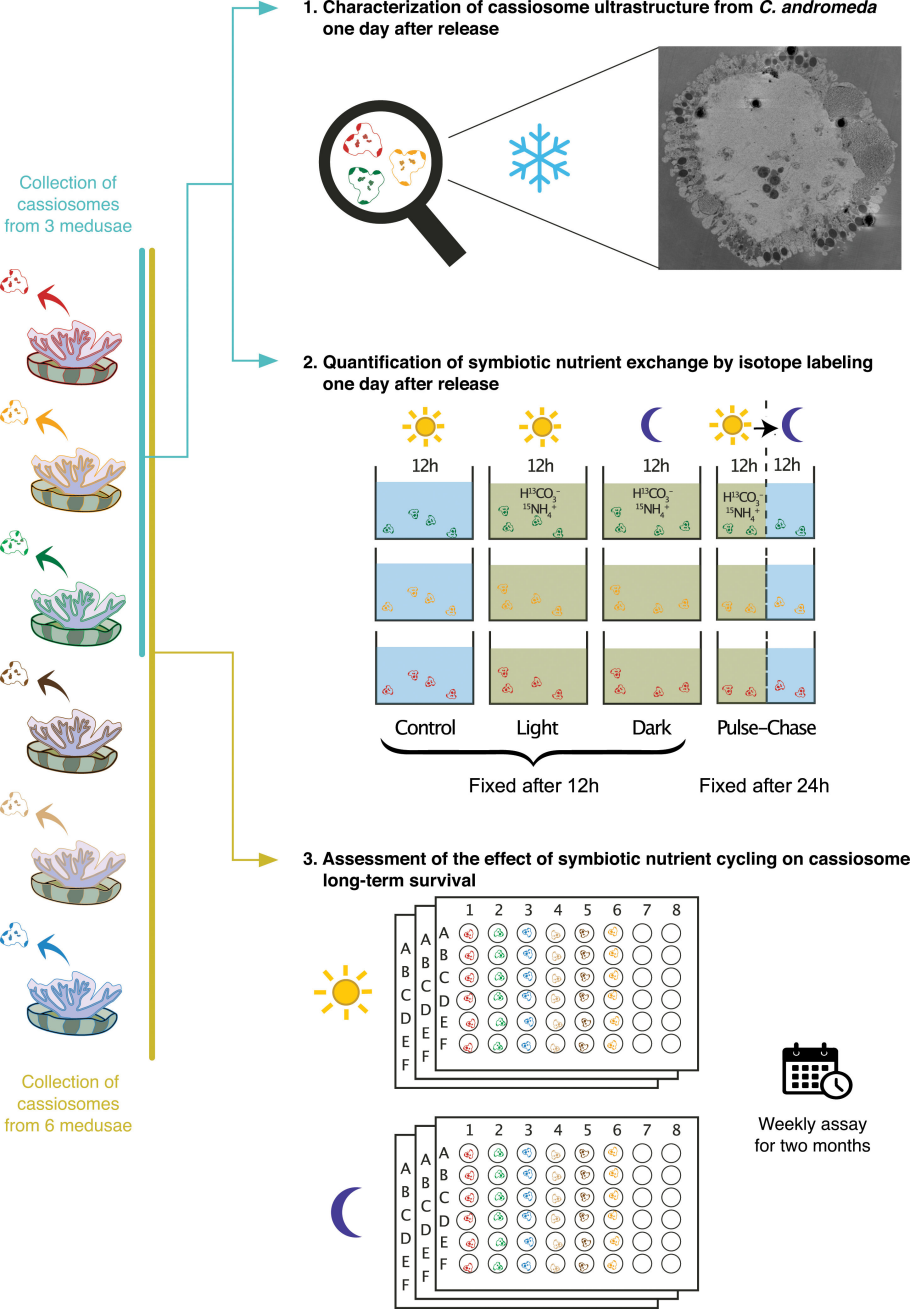
Schematic illustration of the study design composed of three experiments. The colors of cassiosomes in the individual experiments indicate the different medusae they were originally collected from. The number of cassiosomes per beaker in the second experiment is for illustrative purposes only and not a quantitative representation of the actual experiment (cf. Materials and Methods).

In this study, we first describe the ultrastructure of *Cassiopea andromeda* (Forskål, 1775) cassiosomes with a workflow including high-pressure freezing (HPF) and cryo-scanning electron microscopy (cryo-SEM) imaging. We then test the metabolic activity and potential nutritional exchange between cassiosomes and their dinoflagellates by stable isotopic labeling and correlative SEM and nanoscale secondary ion mass spectrometry (NanoSIMS) imaging. Finally, we assessed the contribution of dinoflagellates photosynthates to cassiosomes survival by maintaining cohorts of cassiosomes on either a 12 h:12 h day-night light cycle or in complete darkness in a 2-month-long *in vitro* experiment (Fig. 1).

## MATERIALS AND METHODS

### Animal husbandry and cassiosome collection

Adult *Cassiopea* medusae were acquired from De Jong Marinelife in the Netherlands. Amplification and sequencing of fragments of the COI (mitochondrial cytochrome oxidase subunit I) regions from three individuals (from the same culture system but not used in the experiment) identified the species as *C. andromeda* (GenBank BioSample accession SAMN37108993). The identity of the algal symbiont genus (clade) was previously assessed by amplification of the 28s rRNA gene using pairs of genus/clade-specific primers and the amplification was assessed by gel electrophoresis (25). This revealed that the dominant genus present in the *C. andromeda* medusae culture was *Symbiodinium*. In the 200 L culture aquarium, the medusae were maintained in artificial seawater (ASW) prepared from sea salts (Reef Salt, Aquaforest) at a constant salinity of 35 ppt and temperature of 25°C, illuminated with approximately 100 µmol photons m^−2^ s^−1^ (400–700 nm) from LED lights on a 12 h:12 h day:night cycle. The medusae were fed *ad libitum* two to three times a week with freshly hatched *Artemia salina* nauplii.

For the experiments, cassiosomes were collected from six individual medusae of approximately 5 cm in diameter. Animals were gently sprayed with a jet of ASW in a small beaker to cause the release of cassiosomes. 100 mL of ASW containing cassiosomes was collected from each animal and placed overnight in an incubator at 25°C on a 12 h:12 h day:night cycle, in order to separate the sinking cassiosomes from the floating mucus prior to their use in any of the experiments (19).

### Characterization of cassiosome ultrastructure by cryo-SEM

In order to characterize the ultrastructure of the cassiosomes in their most pristine condition, cassiosomes were fixed, prepared, and imaged using a fully cryogenic workflow (30–32).

The day following their release, cassiosomes were collected by gentle pipetting from the bottom of a petri dish (thus avoiding the floating mucus) using a stereomicroscope, transferred into a 1.5 mL tube, and concentrated by centrifugation at 425 *g* for 2 min. HPF was used for pristine cryopreservation of the cassiosomes. HPF delivers synchronized pressurization and cooling of small samples (<200 µm thick) with liquid nitrogen within 20 ms, thereby avoiding any nucleation of ice crystals that would damage the tissue ultrastructure (30). For this, the pellet of cassiosomes was resuspended in a small volume of the cryoprotectant 20% dextran 40 (prepared in 35 ppt ASW, Sigma D-1662, USA). A small amount of the resuspended cassiosomes was pipetted into an Au-coated Cu-carrier and high-pressure frozen using a Leica EM ICE high-pressure freezer (Leica Microsystems, Germany). Cryopreserved samples were cryo-planed with a diamond trim knife (DiATOME, Switzerland) using a UC7 ultramicrotome (Leica Microsystems, Germany) at −110°C, and transferred to a Leica EM ACE 600 (Leica Microsystems, Germany) for a two-step process. First, freeze etching was performed in order to eliminate any surface ice contamination deposited after trimming and to create morphological contrast between cellular components (31). For this, the sample was warmed up from −150°C to −93°C at a rate of 3°C min^−1^, then held at −93°C for 2 min and brought back down to −150°C at a rate of 3°C min^−1^. Second, a 3 nm platinum layer was deposited by e-beam evaporation at −150°C to minimize surface charging during subsequent cryo-SEM imaging. Finally, the samples were transferred and imaged by cryo-SEM (GeminiSEM 500, Zeiss, Germany; 1.7 kV, aperture size of 10 µm, and a working distance of 3.4 mm) with an Inlens detector (Zeiss, Germany). Cryo-SEM images were adjusted in contrast and brightness, as well as artificially colored for optimized visualization of the structures using Photoshop software (Adobe Photoshop 2023, version 24.3.0).

### Stable isotope labeling experiment

In order to investigate the uptake and exchange of nutrients between the cassiosomes and their Symbiodiniaceae symbionts, a stable isotope labeling experiment was performed using cassiosomes one day after their release from three adult medusae (i.e., three independent biological replicates in total).

The day before the labeling experiment, filtered ASW was depleted of any dissolved organic carbon by acidification with HCl (4 M) to a pH < 3, and maintained under constant air bubbling for at least 4 h. This ASW was then labeled with ^13^C-bicarbonate (#372382, Sigma-Aldrich, USA) to a final concentration of 3 mM. Finally, the pH of the solution was raised again to 8.1 with 1 M NaOH solution and labeled with ^15^N-ammonium-chloride (#299251, Sigma-Aldrich, USA) to a final concentration of 3 µM. After thorough homogenization, the labeled ASW and freshly prepared unlabeled ASW were pre-warmed and maintained at 25°C overnight.

On the morning of the experiment, floating mucus was removed by pipetting off 20 mL of water from the surface of the three beakers containing the cassiosomes. The remaining content in each of the beakers was gently mixed, split into four equal fractions of 20 mL, and concentrated by filtration through a 40 µm cell strainer (Corning, USA). The four fractions of each cassiosome sample were resuspended in 40 mL of labeled or unlabeled ASW accordingly.

Subsequently, cassiosomes collected from each medusa (*n* = 3) were subjected to four different experimental conditions: light, dark, pulse-chase, and control (Fig. 1). To assess the nutrient assimilation by the cassiosomes and their endosymbiont algae with or without photosynthesis, incubations of 12 h in labeled ASW were performed in light and darkness, respectively. In addition, to assess potential relocations over time of the isotopes assimilated during the light period, a pulse-chase experiment was carried out consisting of a 12 h incubation in labeled ASW in light followed by 12 h in unlabeled ASW in darkness. Finally, the remaining cassiosome batches were maintained in unlabeled ASW in the light for 12 h to generate unlabeled control samples with natural isotopic composition of both cassiosomes and algae.

The labeling incubation was performed in glass beakers maintained at 25°C in a 15 L water bath equipped with a circulation pump and a heater. During the incubation, the samples were illuminated for 12 h with LED lights (Viparspectra V165, USA) providing approximately 100 µmol photons m^−2^ s^−1^ (400–700 nm) in the light condition. The dark condition was maintained in constant darkness by wrapping the beakers in aluminum foil. To ensure stable concentrations of the isotope tracers in the incubation water, the water of each beaker was gently mixed every 2 h, and half of the volume of labeled or unlabeled ASW was replaced in each sample every 4 h. At the end of the 12 h incubation, all samples were gently concentrated by filtration using a cell strainer (40 µm mesh size), and the samples corresponding to light, dark, and control conditions were resuspended in 3 mL of fixative solution (4% paraformaldehyde, 2.5% glutaraldehyde and 9% sucrose in 0.1 M Sorensen’s phosphate buffer) for 16 h before further processing. The samples subjected to a pulse-chase were resuspended in unlabeled seawater and incubated for 12 more hours in the dark. At the end of this chase period, the cassiosomes were filtered and chemically fixed as previously described for 4 h.

### Sample preparation for correlative SEM–NanoSIMS imaging and analysis

After fixation, all the samples from the isotope labeling experiment were prepared for correlative SEM and NanoSIMS imaging. Each of the 12 cassiosome samples (derived from four treatments × three source medusae) was split into two aliquots in 1.5 mL Eppendorf tubes and rinsed twice to remove any trace of fixative (centrifugation at 425 *g* for 5 min and rinsed by resuspension in 0.1 M Sorensen’s buffer). To preserve the lipid fraction of the samples, a post-fixation was performed for 1 h with osmium tetroxide (OsO_4_ 1%, 1.5% potassium hexacyanoferrate II in 0.1 M Sorensen's phosphate buffer) under constant agitation, and rinsed by centrifugation at 425 *g* for 5 min and resuspension in milli-Q water under constant agitation for 15 min. After another centrifugation cycle (at 425 *g* for 5 min), the supernatant was discarded and the samples were pre-embedded in agarose to avoid the loss of cassiosomes in the subsequent steps. 20 µL of the cassiosome pellets were transferred into 400 µL polyethylene microtubes (#391178, Milian) pre-filled with 200 µL of 2% liquid agarose at 40°C, and immediately centrifuged at 20,800 *g* for less than 1 min. After curing on ice for 5 min, the tubes were cut open and the agarose-embedded pellets of cassiosomes were dissected into pieces of approximately 1 mm^3^. Using a tissue processor (Leica Microsystems, Germany), the samples were then subjected to serial dehydration in ethanol (30%, 70%, and 100% ethanol in Milli-Q water), to facilitate a progressive Spurr resin infiltration of the samples (30%, 70%, and 100% Spurr resin in absolute ethanol). Once infiltrated, the samples were transferred into molds filled with 100% Spurr resin and cured at 60°C for 48 h. Semi-thin sections (200 nm) of the samples were cut from the resin blocks using an Ultracut S microtome (Leica Microsystems, Germany) and a diamond knife. These sections were then transferred to clean glow-discharged glass slides (for NanoSIMS analysis) or silicon wafers (for correlative SEM and NanoSIMS imaging).

In order to add contrast and to visualize the subcellular structures of the cassiosomes and the algae, the sections on silicon wafers were post-stained with 1% uranyl acetate and Reynolds Lead Citrate before imaging by SEM (Gemini 500, Zeiss, Germany; 3 kV, aperture size of 30 µm, and a working distance of 2.9 to 2.3 mm) with an energy selective backscatter detector (EsB, grid of 130 V; Zeiss, Germany). Prior to NanoSIMS imaging (33), sections were sputter coated with a 12 nm gold layer (using a Leica EM SCD050 gold coater). In the NanoSIMS, the pre-sputtered samples were bombarded with a Cs^+^ primary ion beam at 16 keV with a current of around 2 pA, focused to a spot size of ca. 150 nm. For each image, this beam was rastered over an area of 40 × 40 µm with a resolution of 256 × 256 pixels and a dwelling time of 5,000 µs per pixel for five consecutive layers. The secondary ions ^12^C^12^C^-^, ^12^C^13^C^-^, ^12^C^14^N^-^, and ^12^C^15^N^-^ were counted individually in electron multiplier detectors at a mass resolution power of around 9,000 (Cameca definition), which resolves potential interferences in the mass spectrum. The resulting isotopic maps were analyzed using L’Image (v.10-15-2021, developed by Dr. Larry Nittler, Arizona State University). Images were drift corrected and regions of interest (ROIs) were drawn around different compartments, i.e., dinoflagellates, cassiosome amoebocytes (excluding the dinoflagellates), and the cassiosome epidermis. For each ROI, the isotopic ratio enrichments established through the ratios ^12^C^13^C^-^/^12^C_2_^-^ and ^15^N^12^C^−^/^14^N^12^C^−^ were quantified against a control sample with natural isotopic compositions prepared and analyzed in an identical manner. Isotope enrichments are expressed in the delta notation as follows:


$$\begin{array}{rl} & \delta^{13}C(‰)=\left(\left(\frac{rC(\text{ sample })}{rC(\text{ control })}\right)-1\right)\times 1000\\ & \text{ and }\\ & \delta^{15}N(‰)=\left(\left(\frac{rN(\text{ sample })}{rN(\text{ control })}\right)-1\right)\times 1000 \end{array}$$

where rC_(sample)_ and rC_(control)_ are the count ratios of ^12^C^13^C^-^/^12^C_2_^-^ in the sample and the unlabeled control, respectively. rN_(sample)_ and rN_(control)_ are the count ratios of ^15^N^12^C^−^/^14^N^12^C^−^ in the sample and the unlabeled control, respectively. A compartment was only considered to be isotopically enriched if its average delta-value was more than three standard deviations above the average ratio measured in similar compartments in the unlabeled sample.

### Long-term survival experiment

To investigate the contribution of symbiont photosynthesis to the long-term survival of these autonomous structures, cassiosomes from six adult medusae were maintained in a light-dark cycle or constant darkness for two months (Fig. 1). One day after being released, cassiosomes (36 per medusa) were distributed over six sterile flat-bottom 48 well plates (one cassiosome per well, adding up to a total of 216 cassiosomes; Costar 3548, Corning, USA) in 1.5 mL of filtered-sterilized ASW (filtered through 0.22 µm pore size) at a salinity of approximately 35 ppt. Cassiosomes selected for the experiment were individually observed under a stereomicroscope equipped with a blue light and GFP filter (M165 C, Leica Microsystems, Germany) to verify their motility and the presence of pigmented algal symbionts (by fluorescence). Each plate was then placed into a humid chamber (sealed in a transparent zip-lock bag containing wet tissue paper to avoid evaporation). All six humid chambers containing the cassiosomes in 48 well plates were maintained for two months in an incubator at a constant temperature of 25°C. Three replicate chambers were maintained on a 12 h:12 h light:dark cycle at approximately 100 µmol photons m^−2^ s^−1^ (400–700 nm), and three were maintained in constant darkness (wrapped in aluminum foil and kept in a separate dark compartment in the incubator). The presence/absence of each of the 216 cassiosomes was individually assessed by stereomicroscopy on a weekly basis for a duration of 8 weeks, as an estimation of cassiosome survival. A total of 66% of the ASW in each well was also carefully replaced by pipetting every week. On the last day of the experiment, the presence of dinoflagellates in cassiosomes was qualitatively assessed by fluorescence microscopy, and images of representative cassiosomes of each treatment were acquired by stereomicroscopy.

### Statistical analysis

All statistical analyses were performed in R (version 4.2.0, (34)). The difference in isotopic enrichment between experimental conditions was analyzed using a linear mixed model (LMM) with the medusa of origin as a random variable. This analysis was followed by a Tukey’s Honestly Significant Differences (HSD) post hoc comparison. The overall impact of light and time on the cassiosome estimated survival was analyzed for the linear phase of the response (from day 0 to 35) using a LMM with the medusa of origin as a random variable. The difference between light treatments on each day was then analyzed by a pairwise *t*-test with a subsequent Bonferroni correction of the *P*-values.

## RESULTS

### Description of the *C. andromeda* cassiosome ultrastructure by light microscopy and cryo-SEM

The cassiosomes collected from *C. andromeda* showed considerable variation in size and shape and exhibited motility that can be attributed to the presence and movement of cilia. Most, but not all, of the collected cassiosomes harbored Symbiodiniaceae.

**FIG 2:**
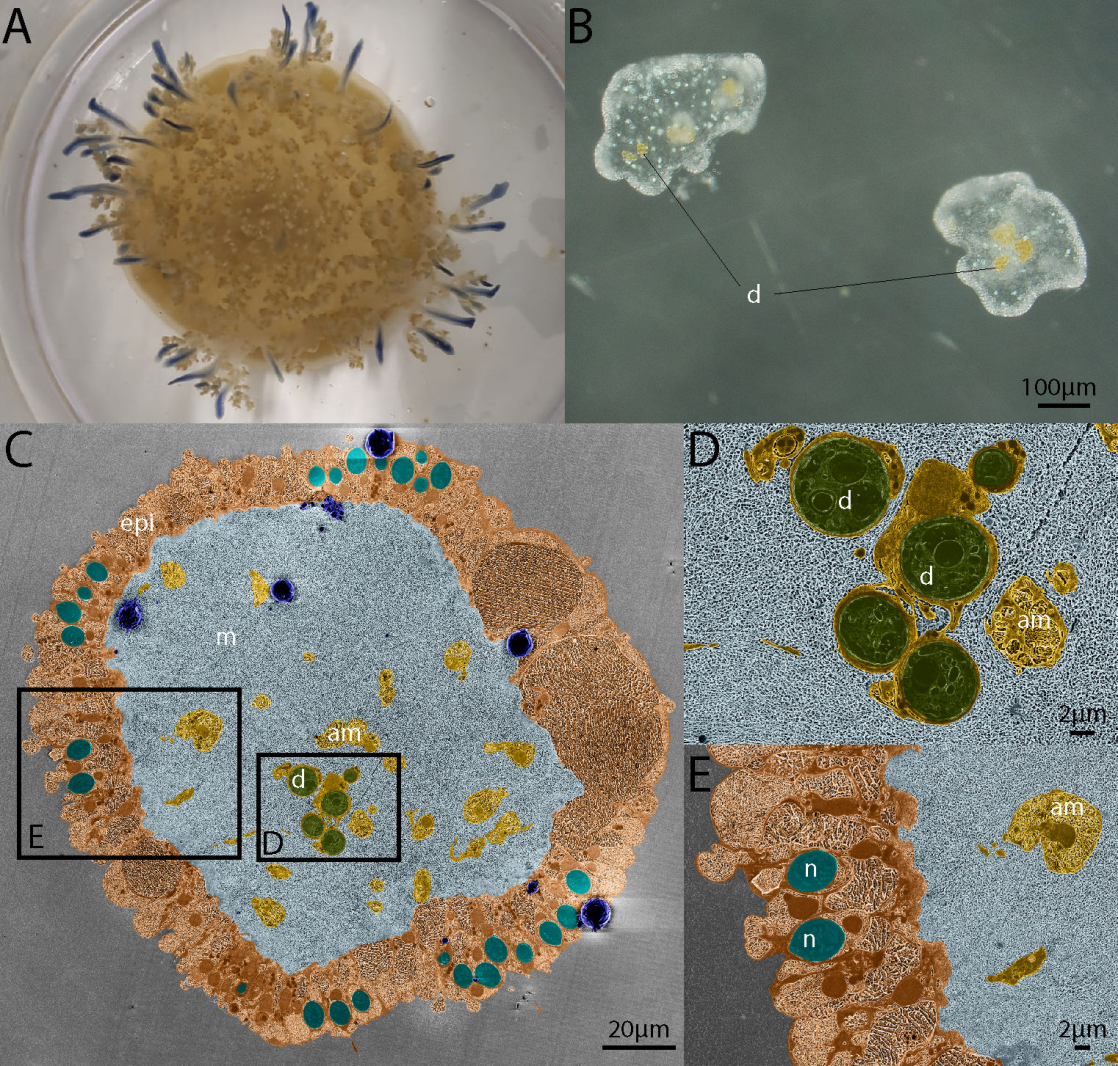
Appearance and ultrastructure of cassiosomes. (A) Adult *Cassiopea andromeda* medusae producing cassiosomes (approximately 5 cm bell diameter). (B) Appearance of freshly collected cassiosomes. (C) Cross-section of a representative cassiosome harboring dinoflagellates imaged by cryo-SEM. (D) Ultrastructural details of amoebocytes hosting dinoflagellates and (E) ultrastructural details of the cassiosome epidermis harboring nematocytes in a representative cassiosome imaged by cryo-SEM. Cryo-SEM images are artificially colored for better visualization of cassiosomes features: d: dinoflagellate (in green), epi: cassiosome epidermis (in beige), m: mesoglea (in light blue), am: amoebocyte (in yellow), n: nematocyst (in turquoise). Ice crystal surface contamination is highlighted in dark blue.

Cryogenic imaging permitted the observation of ultrastructural features of the cassiosomes in their most pristine condition (Fig. 2). Overall, the cassiosomes consisted of an external epidermal cell layer surrounding a “core” of mesoglea. The epidermal cell layer contained a high density of nematocytes, often grouped in clusters (Fig. 2C and E). Inside the mesoglea core, amoebocyte cells were present, some of which were hosting dinoflagellates (Fig. 2C and D).

### Nutrient assimilation and exchange in the cassiosomes and their algal symbionts

**FIG 3:**
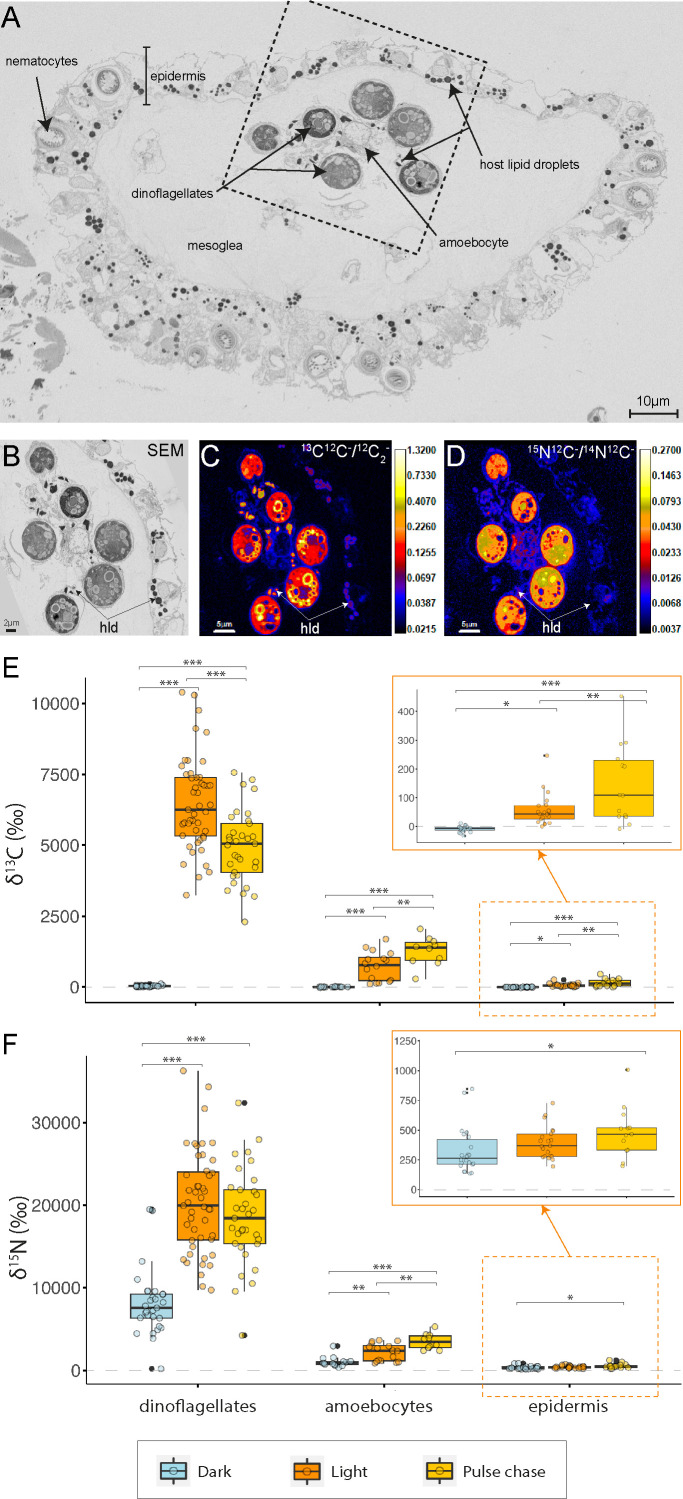
Assimilation of inorganic carbon and nitrogen and translocation of their metabolic derivatives within the cassiosome-algal symbiosis. SEM image of a cross-section of a representative resin-embedded cassiosome (**A**) illustrating its cellular organization. Correlative SEM (**B**) and NanoSIMS (**C, D**) imaging showing the subcellular localization of ^13^C assimilated from ^13^C-bicarbonate (**C**) and ^15^N assimilated from ^15^N-ammonium (**D**) in light (NanoSIMS images are shown with isotope ratios expressed in logarithmic color scale). hld: host lipid droplets. Quantification of ^13^C enrichment (**E**) and ^15^N enrichment (**F**) in cassiosome compartments (dinoflagellates, amoebocytes, and epidermis) under different experimental conditions. Asterisks indicate significant differences between treatments (*P < 0.050, **P < 0.010, ***P < 0.001).

The correlative SEM–NanoSIMS analysis of the ^13^C-bicarbonate and ^15^N-ammonium labeling experiment revealed active and light-dependent assimilation and translocation of nutrients within the cassiosome-algal symbiosis (Fig. 3). After the 12 h incubation in the light, the three measured cassiosome compartments (i.e., dinoflagellates, amoebocytes, and epidermis) were significantly enriched in ^13^C (Fig. 3B, C, and E). This ^13^C enrichment was particularly apparent in the starch granules of the dinoflagellates and the abundant lipid droplets (dark intracellular bodies stained by osmium in SEM images) present in the amoebocytes and epidermis (Fig. 3B and C). Similarly, all compartments were enriched in ^15^N (Fig. 3F). The ^15^N enrichment was distributed quasi-homogeneously within the dinoflagellate cells, the amoebocytes, and the epidermis cells, respectively (Fig. 3B and D).

The absence of light during the 12 h incubation strongly impacted nutrient assimilation in the cassiosomes (Fig. 3E and F). In the dark, the ^13^C enrichment was undetectable (below the enrichment threshold) in the dinoflagellates, amoebocytes, and epidermis (Tukey’s HSD, *P* < 0.001 for dinoflagellates and amoebocytes, *P* = 0.022 for the epidermis, Fig. 3E). The ^15^N enrichment was also significantly lower in the dark compared to the light condition, specifically by 58% in the dinoflagellates (Tukey’s HSD, *P* < 0.001), 54% in the amoebocytes (Tukey’s HSD, *P* = 0.003) and 19% in the epidermis (albeit not significantly; Tukey’s HSD, *P* = 0.273, Fig. 3F).

Finally, the light pulse followed by a dark chase period (pulse-chase condition) revealed the temporal cascade of nutrient assimilation and translocation in the symbiosis. Compared to the light condition (i.e., pulse without a chase period), the ^13^C enrichment in the dinoflagellates decreased significantly by 25% over the subsequent 12 h dark period (Tukey’s HSD, *P* < 0.001). In contrast, the ^13^C enrichment increased by 67% in the amoebocytes (Tukey’s HSD, *P* = 0.004) and by 145% in the epidermis (Tukey’s HSD, *P* = 0.003, Fig. 3E). In addition, while the ^15^N enrichment remained overall similar following the 12 h dark chase in the dinoflagellates (Tukey’s HSD, *P* = 0.828), it experienced an increase of 56% in the amoebocytes (Tukey’s HSD, *P* = 0.002) and 36% epidermis (Tukey’s HSD, *P* = 0.141, Fig. 3F) after the next 12 h dark period, when compared with the light condition.

### Light enhances cassiosome survival

**FIG 4:**
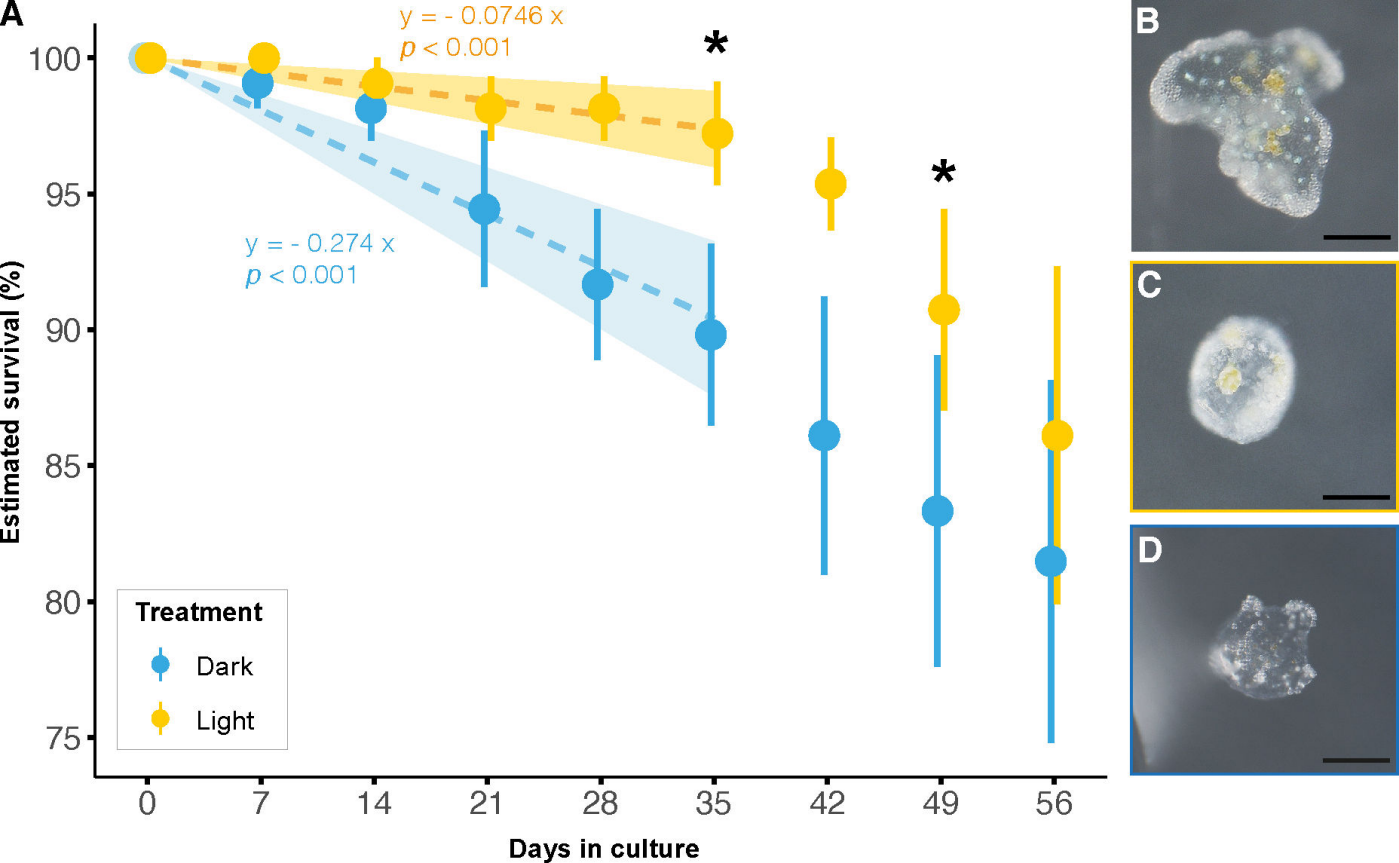
Influence of light treatments on cassiosome survival. (A) Estimated survival of cassiosomes over time *in vitro*, under a 12 h:12 h light:dark cycle (yellow) or in constant darkness (blue). Filled circles and error bars indicate the mean ± SE. *n* = 108 cassiosomes from a total of six different medusae were used per condition. Asterisks indicate statistically significant differences between treatments (*P < 0.050) at a given time point. Images of chemically fixed cassiosomes before (**B**) or after being maintained in light (**C**) or dark (**D**) conditions for 2 months (scale bar = 100 µm).

The 2-month-long culture experiment illustrated the impact of light availability on symbiont-bearing cassiosome survival *in vitro* (Fig. 4). Overall, the interaction of light treatment and time had a significant effect on cassiosome survival (LMM, *X^2^* = 13.46, *P* < 0.001, Fig. 4A) during the first 35 first days of the experiment. During this linear phase of the cassiosome decline, the rate of disappearance of cassisomes in the dark was 3.6-fold higher than in the light. Over time, this led to a significant difference in survival between treatments (pairwise *t*-tests, *P-adjusted* = 0.043, 0.025 for day 35 and 49 respectively, Fig. 4A). After day 35, the rate of disappearance of cassiosomes in the light increased and exceeded the disappearance rate of the dark condition.

Of note, more than 80% of the cassiosomes were still present in both conditions at the end of this two-month experiment, but their appearance changed. The cassiosomes experienced a strong reduction in size, often losing their characteristic “popcorn”-like shape, and became featureless (Fig. 4B, C, and D). While no quantification has been performed, individual cassiosomes (not the entire well) were inspected for chlorophyll fluorescence at the end of the experiment using a stereomicroscope: the dinoflagellates in most of the cassiosomes kept in the light condition were still fluorescent, while most of the cassiosomes kept in the dark condition showed no chlorophyll fluorescence.

## DISCUSSION

Global warming and local anthropogenic stressors, such as overfishing and eutrophication, have been linked to recent local increases in jellyfish population density, spatial distribution, and stinging threat (4, 7). Some species of the upside-down jellyfish *Cassiopea* have been described as particularly invasive in several tropical and subtropical regions around the world in recent years (11–13). Several *Cassiopea* species and other members of the Rhizostomeae order have been shown to release stinging, autonomous, and often motile tissue structures called cassiosomes. These cassiosomes are likely a major contributor to the “contactless” stinging phenomenon (18, 19). Here, we showed that dinoflagellates act as beneficial symbionts within the cassiosome by fueling their host’s metabolism with photosynthates and thus prolonging their autonomous life span.

### The ultrastructure of cassiosomes from *C. andromeda*

The ability of *C. andromeda*, another rhizostome medusa species, to also produce cassiosomes supports the idea of them being a ubiquitous evolutionary feature of the Rhizostomeae order (19). The cassiosomes of *C. andromeda* were composed of an external epidermis containing a high number of nematocytes, and a core of mesoglea harboring amoebocytes that frequently hosted dinoflagellates (Fig. 2). This overall cellular organization is similar to the one previously described for the cassiosomes of *C. xamachana* (19).

### Algal symbionts contribute photosynthates to the cassiosome metabolism

One day after their release by medusae, the cassiosomes incubated with ^13^C-bicarbonate and ^15^N-ammonium in light showed strong enrichments in ^13^C and ^15^N in cassiosome cells (amoebocytes and epidermis) and their dinoflagellates (Fig. 3). This demonstrates that cassiosomes are anabolically active and able to assimilate nutrients from the seawater, even after their physical separation from the medusa. In the dark, the disappearance of ^13^C enrichment (Fig. 3E) indicates that the fixation of inorganic carbon from the seawater is primarily driven by dinoflagellate photosynthesis in a light-dependent manner. The associated drop in ^15^N enrichment in the cassiosome cells (Fig. 3F) is likely caused by a reduction in carbon availability in the cassiosomes in the absence of algal photosynthesis. Indeed, cnidarian ammonium assimilation requires the availability of excess carbon backbones in the TCA cycle for amino acid synthesis.

Consistently with this, following the 12 h pulse labeling in the light, a 12 h dark chase period with unlabeled ASW caused a decrease in ^13^C enrichment in the dinoflagellates and an increase in ^13^C enrichment in amoebocytes and epidermis (Fig. 3E). This indicates that an active transfer of photosynthetically fixed carbon took place from dinoflagellates to the cassiosome tissues. This increase of ^13^C enrichment was associated with an increase in ^15^N assimilation in the cassiosome tissue (Fig. 3F), again best ascribed to organic carbon availability in the cassiosome tissue: as carbon availability increases with time in the cassiosome tissue through the translocation process, the cassiosome cells can anabolically assimilate more ammonium.

In conclusion, these results demonstrate that cassiosomes maintain an active metabolism that is supported by symbiotic nutrient exchange with their associated dinoflagellates. The algal symbionts fuel and shape the metabolism of cassiosomes with photosynthetically fixed carbon, in a manner highly similar to the symbiotic interactions observed in the medusa (25) and other marine photosymbioses (35–40).

### Symbiotic nutrient input supports the long-term survival of cassiosomes *in vitro*

The positive impact of light and associated photosynthetic input from dinoflagellate symbionts on cassiosome survival was reflected in a significantly higher survival rate during the first five weeks of the *in vitro* experiment, compared with the dark condition (Fig. 4). While light (and associated algal photosynthesis) enhanced cassiosome survival until day 35 relative to the dark condition, this advantage diminished after that day. Overall, it is plausible that cassiosomes in the light eventually become limited in some other essential nutrients (e.g., nitrogen).

At the same time, the survival of most cassiosomes in both treatments for two months suggests that cassiosomes do not entirely rely on symbiont photosynthesis for their carbon requirements. The abundance of lipid droplets visualized by SEM in the amoebocytes and the epidermis, coupled with a carbon-rich mesoglea core (Fig. 3A), may represent another important source of carbon that can support the metabolic requirements of cassiosomes. The here-described decrease in cassiosome size and appearance throughout the experiment (also previously described in *C. xamachana* (19)), thus likely reflects the gradual depletion of energy reserves in the cassiosomes. Hence, the life span of autonomous cassiosomes may in part depend on their initial energy reserves. In our study, cassiosomes showed high initial energy reserves as reflected in the abundance of lipid droplets (inherited from their regularly-fed medusae). Combined with the stable and microbially depleted *in vitro* condition, this likely contributed to the longer survival period of the cassiosomes in this study compared to the 10 days previously reported for those of *C. xamachana* (19). However, our results highlight the surprisingly long-lived metabolic capacity of these small autonomous tissue structures. Further studies may investigate the survival and stinging capacity of cassiosomes over time in more natural environments.

### Ecological relevance

Our study indicates that the presence of symbiotic dinoflagellates in cassiosomes can increase their autonomous lifetime in the water column. In this context, Anthony et al. (20) previously reported that the presence of dinoflagellates in cassiosomes of *C. ornata* differed between locations, and suggested that this difference could reflect different levels of investments in heterotrophic feeding. The differences in dinoflagellate abundance in cassiosomes could be due to differences in host dinoflagellate population densities and/or in the frequency of cassiosome release relative to the algal symbiont division rate. In any case, our results suggest that the presence of dinoflagellates in cassiosomes may sustain and actually enhance their autonomous life span, thereby indirectly enhancing the heterotrophic feeding capacities of *Cassiopea* medusae.

Considering the importance of the algal symbionts in the life cycle of *Cassiopea,* and the specificity of this symbiotic interaction (41–44), it is surprising that *Cassiopea* produces only aposymbiotic larvae, which requires horizontal acquisition of dinoflagellates and selection of homologous symbionts by the polyp (45). In this context, it is possible that cassiosomes might constitute an environmental “reservoir” of homologous symbionts in the environment, thereby facilitating symbiotic establishment for the newly formed polyps.

### Cassiosomes, a miniaturized model system for the cnidarian-Symbiodiniaceae symbiosis?

This study has shown that freshly released cassiosomes are metabolically active miniaturized holobionts that can effectively assimilate, exchange, and recycle nutrients autonomously. In particular, the symbiotic interface of the amoebocyte-dinoflagellate association seems to behave in a manner similar to other photosymbiotic cnidarians (such as corals), making cassiosomes a powerful laboratory model system for cell-to-cell symbiotic interactions (cell recognition, nutritional exchanges, etc.). Their year-round availability and high abundance, easy collection process, and relatively simple structural organization may provide advantages over the use of symbiotic polyps, larvae, or entire adult specimens of Cnidaria. Their small size falls well within the technical limits of pristine vitrification by HPF (around 200 µm, (46)) making them particularly suitable for in-depth characterization of cellular ultrastructure (e.g., using cryo-SEM) and Cryo-NanoSIMS isotopic imaging (32). Studies of symbiotic interactions and the protein composition of the symbiosome (i.e., with whole-mount immunolabeling experiments) also seem possible within these interesting “tissue balls”. Even though the observed range in cassiosome shape, size, and dinoflagellate abundance might be a source of experimental variability, the preservation of symbiotic nutrient exchange in these small cellular structures makes cassiosomes an attractive and powerful new miniaturized model system for the detailed study of the interface and machinery of cnidarian photosymbiosis.

## Data Availability

All raw data associated with this study have been deposited in the zenodo.org repository.
